# Trends and determinants of tuberculosis incidence in Turkey: A secondary data analysis

**DOI:** 10.1097/MD.0000000000048278

**Published:** 2026-04-17

**Authors:** Yusuf Demirtaş, Turgut Şahinöz

**Affiliations:** a Department of Public Health, Faculty of Medicine, Ordu University, Ordu, Turkey; b Department of Health Management, Faculty of Health Sciences, Ordu University, Ordu, Turkey.

**Keywords:** Human Development Index, incidence, social determinants of health, tuberculosis, Turkey

## Abstract

Tuberculosis remains a major public health concern, with incidence rates influenced by social determinants of health. Although tuberculosis incidence in Turkey has declined markedly in recent decades, the factors associated with this decline have not been comprehensively evaluated at the country-level. This study aimed to examine trends in tuberculosis incidence in Turkey from 1982 to 2021 and to identify determinants of tuberculosis incidence rates between 2000 and 2021, considering variables categorized under composite development, economic indicators, population, and health services. A longitudinal ecological study was conducted using secondary data to examine trends and determinants of tuberculosis incidence in Turkey. Univariate and multivariable linear regression were employed to evaluate the associations between tuberculosis incidence rates and 10 selected variables. Tuberculosis incidence in Turkey declined substantially over the 40-year period analyzed, with an overall decrease of 86.6% and sharper reductions observed in certain intervals. In multivariable linear regression, only the Human Development Index remained independently associated with tuberculosis incidence (β = −1.041, *P* < .001), indicating that higher Human Development Index values were associated with lower tuberculosis incidence rates in Turkey. These findings indicate that tuberculosis incidence in Turkey has declined alongside changes in human development, as reflected by the composite Human Development Index.

## 1. Introduction

Tuberculosis (TB) remains a major global public health concern, affecting over 10 million people annually and is the leading cause of death from a single infectious agent.^[1]^ Although advances such as chemotherapy, short-course treatment, and effective control programs have reduced TB incidence globally, the incidence rates vary substantially between regions, with the World Health Organization (WHO) European Region reporting <30 cases per 1,00,000 population/yr, compared to over 200 per 1,00,000 in South-East Asia Region and African Region.^[1,2]^

Despite improvements in case detection and treatment, TB incidence has not declined as rapidly as expected.^[3]^ Recent studies highlight the role of social determinants of health in explaining TB incidence rates.^[4-9]^ Studies have reported that the national Human Development Index (HDI) is one of the main predictors of TB incidence, particularly in low- and lower-middle-income countries.^[5,6]^ Other factors, including socioeconomic, demographic, and health system indicators, have also been shown to be associated with TB incidence rates, although their effects vary across countries and regions.^[3-6,8]^ However, there is a notable gap in the literature regarding comprehensive, country-level analyses of tuberculosis incidence determinants in Turkey that apply a consistent methodological framework and incorporate context-specific factors.

Turkey, the second most populous country in the WHO European Region with a population exceeding 85 million, experienced a high TB burden in the early to mid-20th century.^[10]^ In subsequent decades, advances in TB control, together with positive changes in key social determinants of health, were accompanied by a substantial decline in TB incidence.^[11,12]^ However, the relative contribution of these factors to the observed decline remains unclear. Moreover, after 2000, Turkey faced a major financial crisis as well as a mass migration due to the civil war in neighboring Syria, which could have important implications for TB control.^[13-15]^ Tuberculosis is strongly associated with poverty and socioeconomic vulnerability, making periods of economic instability particularly consequential for TB epidemiology.^[16]^ In addition, refugee populations often experience substandard living conditions and increased exposure risks, which may affect both displaced populations and host communities.^[14]^ In the present study, these socioeconomic processes are also represented through corresponding macro-level economic and demographic indicators, rather than being analyzed as discrete shock events. In this context, we consider that assessing TB trends and determinants in Turkey is very important for understanding the dynamics of TB. This assessment may also contribute to the evidence base supporting broader public health discussions related to tuberculosis control.

The study had 2 main objectives. First, we examined long-term trends in tuberculosis incidence rates in Turkey over a 40-year period (1982–2021) to describe changes at the national level. Second, focusing on the period 2000 to 2021, when standardized, comparable, and reliable data are available, we assessed the association between TB incidence rates and selected economic, demographic, and health system indicators in order to identify the key determinants of TB incidence in Turkey.

## 2. Materials and methods

This study used secondary data for analysis. A longitudinal ecological design was employed to assess associations between TB incidence rates and 10 selected variables. Annual TB incidence rates in Turkey were obtained from 2 sources. One source was the World Health Organization’s Global Tuberculosis Report estimates of national TB incidence rates, calculated as the estimated number of new and relapsed TB cases arising in a given year per 1,00,000 population.^[17]^ These estimates were available from 2000, and the updated February 2024 versions of the TB incidence rate estimates for 2000 to 2021 were included in the study. The Ministry of Health’s Health Statistics Yearbooks were used as a second source for an assessment including TB incidence rates from previous years.^[18-20]^ The number of TB cases reported annually (sum of new and relapsed cases) was recorded. Incidence rates were calculated by dividing the number recorded by the country’s mid-year population (according to the United Nations World Population Prospects 2022 estimates) for the relevant year.^[21]^ The more recent yearbook was used as the reference when there were differences in the number of TB cases between yearbooks. TB incidence rates calculated using this method were also analyzed for the period 1982 to 2021. All incidence rates are expressed per 1,00,000 people.

The change in TB incidence rates between 5-year periods was calculated separately using incidence rates derived from both sources. First, the average incidence rates for each period were calculated by dividing the sum of the annual incidence rates by 5. The change in TB incidence rate between 5-year periods was then expressed as a percentage using the formula ((*B*/*A* − 1) × 100), where *A* is the average TB incidence rate in the previous period and *B* is the average TB incidence rate in the corresponding period. The same calculation was also conducted for the world, based on the global TB incidence rate estimates from the WHO’s Global Tuberculosis Report.

The link between TB incidence rates and 10 variables under the headings of composite development, economic indicators, population, and health services was investigated. Following a comprehensive literature review, variables that could potentially determine the TB incidence rate in Turkey, for which sufficient, reliable, and accessible data were available, were included in the study. Data for the independent variables were obtained from 4 sources: the Human Development Reports, the World Bank DataBank, the World Population Prospects, and the Ministry of Health’s Health Statistics Yearbooks.^[18,21-23]^ In this analysis, only national TB incidence rate estimates from the WHO Global Tuberculosis Report were used as the dependent variable because of their reliability and standardization. WHO TB incidence estimates are derived using standardized methodologies that incorporate country-specific adjustments for underreporting, underdiagnosis, and overdiagnosis, thereby improving both temporal comparability and the validity of incidence estimates. In contrast, TB incidence rates calculated from the Ministry of Health’s Health Statistics Yearbooks are based on routinely reported case notifications. Although the national yearbook data provide longer historical coverage, the WHO estimates were selected as the dependent variable as they provide a more standardized and methodologically robust representation of TB incidence for analytical purposes.^[24]^ The data source and years of data availability for each variable are shown in Table 1.

**Table 1:** Data source and years of data availability for each variable used in the analysis to identify determinants of tuberculosis incidence rate in Turkey.

Variable	Data source	Years of data availability
Tuberculosis incidence rate (per 1,00,000 people)	WHO, Global Tuberculosis Report	2000–2021
Composite development		
Human development index	Human Development Reports	2000–2021
Economic indicators		
GDP per capita (PPP, current international $)	World Bank DataBank	2000–2021
Poverty headcount ratio at national poverty lines (% of population)	World Bank DataBank	2005–2020
Inflation of consumer prices (annual %)	World Bank DataBank	2000–2021
Current health expenditure per capita (PPP, current international $)	World Bank DataBank	2000–2020
Proportion of population spending more than 10% of household consumption or income on out-of-pocket health care expenditure (%)	World Bank DataBank	2002–2019
Population		
Population growth rate (%)	World Bank DataBank	2000–2021
Net migration (millions of people)	World Population Prospects 2022	2000–2021
Health services		
BCG vaccination rate (%)	Health Statistics Yearbooks	2000–2021
Tuberculosis treatment success rate (% of new cases)	World Bank DataBank	2000–2021

BCG = bacille Calmette-Guerin, GDP = gross domestic product, PPP = purchasing power parity, WHO = World Health Organization.

Tuberculosis incidence rates (per 1,00,000 population) were evaluated as the dependent variable. Because incidence rates showed a decreasing but nonlinear trend, we applied a log-transformation to stabilize variance and improve linear model fit. Linearity and variance assumptions were assessed by inspection of plots of standardized residuals versus standardized predicted values. Compared with the untransformed scale, the log-transformed specification showed reduced curvature and a more constant spread of residuals across predicted values, indicating improved linearity and homoscedasticity and, consequently, a better-fitting linear model. Associations between TB incidence and each independent variable were first examined using univariate linear regression. Variables showing a *P*-value < .20 in univariate analysis were considered candidates for multivariable modeling. Given the longitudinal structure of the dataset (2000–2021), we initially considered applying time series models. However, the monotonic downward trend in TB incidence, along with the strong correlation of year with HDI and economic indicators, limited this approach. Specifically, year and the HDI were almost perfectly correlated, and including both in the same model would have introduced severe multicollinearity and inflated variance. Since HDI is a composite measure capturing multiple dimensions of development, we retained HDI as the preferred indicator and excluded year to avoid multicollinearity. HDI was prioritized as it represents the multifaceted structural improvements in development more effectively than a simple temporal variable such as year. Additionally, HDI, GDP per capita, poverty headcount ratio at national poverty lines, and current health expenditure per capita were highly correlated (*r* > 0.8) in univariate analyses. To address this, HDI was chosen as the representative variable due to its higher explanatory power and composite nature. Conceptually, HDI provides a more comprehensive reflection of the social determinants of TB, as it captures the interplay between income, educational attainment, and life expectancy. This choice ensures that the model reflects holistic human development rather than focusing on a single economic dimension. In the final multivariable model, HDI, BCG vaccination rate, inflation of consumer prices, and the proportion of population spending more than 10% of household consumption or income on out-of-pocket health expenditure were included. Given the limited number of observations, the number of predictors in the multivariable model was carefully restricted to minimize the risk of overfitting. Multivariable linear regression was conducted, and model assumptions was evaluated using a set of standard diagnostic measures. Overall model fit was assessed using the coefficient of determination (*R*^2^) and the ANOVA *F*-test. Multicollinearity was assessed with variance inflation factors (VIF), with values below 5 considered acceptable. Residual autocorrelation was assessed using the Durbin–Watson statistic, with values between 1.5 and 2.5 indicating no meaningful autocorrelation. For both univariate and multivariable analyses, years with missing values for a given variable were excluded from the respective analysis, and no interpolation or imputation was performed. In the multivariable analysis, one of the included independent variables had missing data in the first and last 2 years of the study period; therefore, the multivariable model was restricted to the 18 years (2002–2019) for which complete data for all included variables were available. Due to the ecological design of the study, all analyses were conducted at the population level, and associations observed between TB incidence rates and variables reflect aggregate, not individual-level relationships. All statistical analyses were performed using IBM SPSS Statistics 24.0 (IBM Corp., Armonk). Statistical significance was inferred at a 2-sided *P*-value < .05.

To further assess the robustness of the findings and address potential time dependence, additional sensitivity analyses were conducted. First, the log-transformed tuberculosis incidence rate and independent variables were transformed into first differences, and multivariable regression analysis were reestimated to examine year-to-year changes. Second, an alternative temporal adjustment was applied by including a period indicator variable distinguishing between an earlier (2000–2010) and a later period (2011–2021) in the multivariable regression model. These sensitivity analyses were performed to evaluate the stability of the main findings under alternative temporal specifications.

## 3. Results

Tuberculosis incidence rate in Turkey declined over the study period. Based on data from the Ministry of Health’s Health Statistics Yearbooks, TB incidence decreased by 86.6% between 1982 and 2021, from 79.7 to 10.7 per 1,00,000 population. According to the WHO Global Tuberculosis Report estimates available for 2000 to 2021, TB incidence declined from 33 to 14 per 1,00,000 population, corresponding to a 57.6% reduction over this period. In multivariable regression analysis, only the HDI remained independently associated with TB incidence, indicating an inverse association.

**Figure 1.**
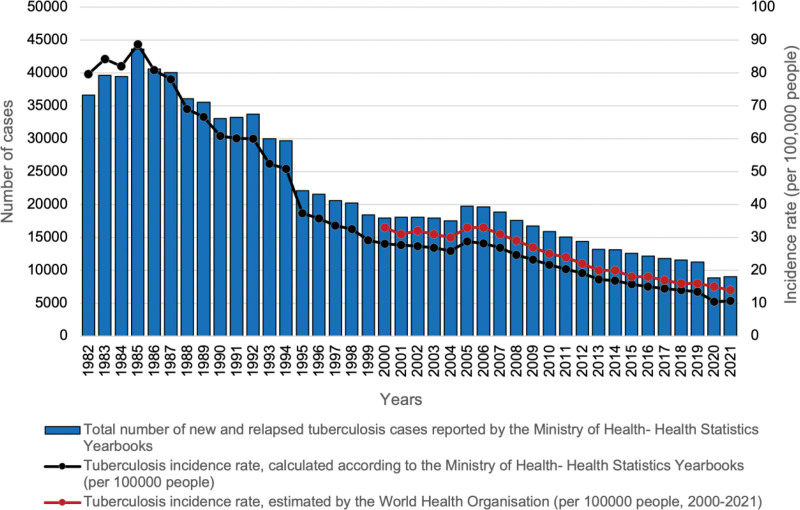
Total number of new and relapsed tuberculosis cases and tuberculosis incidence rates in Turkey between 1982 and 2021.

Figure 1 shows the long-term decline in TB incidence rates in Turkey, with separate lines for national yearbook data and WHO estimates. Despite minor year-to-year fluctuations around this downward trend, incidence rates derived from the 2 sources followed parallel temporal patterns, with WHO estimates consistently higher. The total number of new and relapsed TB cases and TB incidence rates in Turkey between 1982 and 2021 are shown in Figure 1.

Average TB incidence rates calculated for 5-year periods between 1982 and 2021 demonstrates a steady decline. The largest decrease was observed in 1997 to 2001, followed by the smallest reduction in the subsequent 5-year period. During periods with comparable data, Turkey consistently had lower TB incidence rates than the global average, and the decline in each 5-year period was greater in Turkey compared with declines at the global level. Table 2 presents the average TB incidence rates and the changes in incidence rates for 5-year periods in Turkey and globally.

**Table 2:** Average tuberculosis incidence rates and incidence rate changes for 5-year periods between 1982 and 2021.

Years	Average incidence rate, Turkey*^,^‡	Incidence rate change, Turkey†^,^‡	Average incidence rate, Turkey*^,^§	Incidence rate change, Turkey†^,^§	Average incidence rate, world*^,^§	Incidence rate change, world†^,^§
1982–1986	83.1	**–**	**–**	**–**	**–**	**–**
1987–1991	66.4	−20.1%	**–**	**–**	**–**	**–**
1992–1996	47.9	−27.9%	**–**	**–**	**–**	**–**
1997–2001	30.4	−36.5%	**–**	**–**	**–**	**–**
2002–2006	27.4	−9.9%	31.8	**–**	178.6	**–**
2007–2011	23.9	−12.6%	27.2	−14.5%	166.8	−6.6%
2012–2016	16.6	−30.7%	19.6	−28.0%	149.4	−10.4%
2017–2021	12.8	−22.8%	15.6	−20.4%	132.8	−11.1%

* Calculated by dividing the sum of the annual incidence rates by 5 (per 1,00,000 people).

† Calculated according to the average incidence rate of the previous 5-year period.

‡ Based on Ministry of Health – Health Statistics Yearbooks.

§ Based on World Health Organization estimates.

Table 3 presents the univariable associations between TB incidence rate and certain variables in Turkey. Univariate analysis showed that higher HDI, GDP per capita, current health expenditure per capita and BCG vaccination rates were associated with lower TB incidence rates, while higher poverty headcount ratio and proportion of the population spending more than 10% of household consumption or income on out-of-pocket health care expenditure were associated with higher TB incidence rates. Among these significant variables, HDI, GDP per capita, poverty headcount ratio and current health expenditure per capita demonstrated strong explanatory power (*R*^2^ > 0.75). In the multivariable model, HDI was the only determinant retained (β = −1.041, *P* < .001), showing that higher HDI values were associated with lower TB incidence rates in Turkey. Other variables included in the multivariable model were no longer statistically significant. The results of the multivariable regression analysis are presented in Table 4.

**Table 3:** Univariable associations between tuberculosis incidence rate and certain variables in Turkey.

Variable	Min (yr)*	Max (yr)†	Univariate linear regression		
			*B*	*R* ^2^	*P*
Composite development					
Human development index	0.669 (2000)	0.841 (2021)	−2.000	0.950	<.001
Economic indicators					
GDP per capita (thousands $)	9.16 (2001)	30.7 (2021)	−0.017	0.949	<.001
Poverty headcount ratio (%)	13.5 (2016)	18.6 (2005)	+0.074	0.774	<.001
Inflation of consumer prices (%)	6.25 (2009)	54.9 (2000)	+0.003	0.113	.126
Current HE per capita (thousands $)	0.44 (2000)	1.26 (2020)	−0.427	0.869	<.001
Out-of-pocket HE > 10% of income (%)	3.06 (2012)	6.01 (2002)	+0.076	0.303	.018
Population					
Population growth rate (%)	0.76 (2021)	1.99 (2014)	+0.100	0.066	.250
Net migration (millions of people)	−0.26 (2018)	0.70 (2014)	−0.062	0.013	.611
Health services					
BCG vaccination rate (%)	76.0 (2003)	97.0 (2010)	−0.011	0.435	<.001
Tuberculosis treatment success rate (%)	65.0 (2002)	91.0 (2006)	−0.003	0.035	.407

BCG = bacille Calmette-Guerin, GDP = gross domestic product, HE = health expenditure.

* Indicates the lowest value of the variable during the specified period.

† Indicates the highest value of the variable during the specified period.

**Table 4:** Multivariable associations between tuberculosis incidence rate and certain variables in Turkey.

Variable	Multivariable linear regression				
	*B*	95% CI for *B*		β	*P*
Human development index	−2.107	−2.342	−1.871	−1.041	<.001
Inflation of consumer prices	−0.001	−0.002	+0.001	−0.065	.229
Out-of-pocket HE > 10% of income	+0.014	−0.005	+0.032	+0.098	.142
BCG vaccination rate	+0.002	−0.001	+0.005	+0.138	.093

Log-transformation: Tuberculosis incidence rates were log-transformed to stabilize variance and improve linearity.

Multicollinearity: All variance inflation factors (VIF) were below 5 (maximum VIF = 4.00, observed for BCG vaccination rate).

Autocorrelation: Durbin-Watson = 1.682 (within the acceptable 1.5–2.5 range).

Overall model fit: *R*^2^ = 0.981, ANOVA *F*-test *P* < .001.

BCG = bacille Calmette-Guerin, CI = confidence interval, HE = health expenditure, VIF = variance inflation factors.

The results of the sensitivity analyses are presented in Supplemental Digital Content 1, Supplemental Digital Content, https://links.lww.com/MD/R704 and Supplemental Digital Content 2, Supplemental Digital Content, https://links.lww.com/MD/R704. In the first-difference specification, where year-to-year changes in the log-transformed tuberculosis incidence rate and independent variables were examined, none of the variables remained statistically significant in the multivariable model (Supplemental Digital Content 1, Supplemental Digital Content, https://links.lww.com/MD/R704). When an alternative temporal adjustment was applied using a period indicator variable (2000–2010 vs 2011–2021), the direction and magnitude of the associations were similar to those observed in the primary multivariable analysis, while the period indicator itself was not statistically significant (Supplemental Digital Content 2, Supplemental Digital Content, https://links.lww.com/MD/R704).

## 4. Discussion

This study examined the trends in TB incidence in Turkey between 1982 and 2021 and analyzed the determinants of TB incidence in the period 2000 to 2021. It was found that the TB incidence rate in Turkey has significantly decreased over 40 years, with this decline being more pronounced in certain periods. The Human Development Index was consistently associated with TB incidence rates in Turkey.

In the 40-year period analyzed in our study, the annual number of new and relapsed TB cases in the country steadily decreased, with only a few exceptions. The incidence rate, calculated using data from the Ministry of Health’s Health Statistics Yearbooks, increased slightly from 1982 to 1985 and then showed a marked decline until the early 2000s. In the post-2000 period, the decline continued, but the curve began to follow a more horizontal trajectory. The impact of early TB control policies and increased access to health services in the country likely contributed significantly to the rapid decline observed in the first half of the review period. However, after a certain point, the concentration of cases in disadvantaged populations – who are more difficult to detect – and the presence of drug-resistant cases may have slowed the decline. The second source, the WHO Global Tuberculosis Report TB incidence rate estimates, provides reliable and comparable results based on country-specific adjustment methodology.^[24]^ The WHO estimates of annual TB incidence rates for Turkey between 2000 and 2021 show a very similar curve to the incidence rates calculated from local sources, although they present slightly higher values for each year. This discrepancy is related to the WHO’s more comprehensive assessment, which takes unreported cases into account. According to both sources, a similar downward trend in incidence rates is observed between 2000 and 2021.

Before the 2000s, global TB incidence estimates were subject to substantial uncertainty due to the lack of systematic surveillance in many regions. Nevertheless, available evidence suggests marked regional heterogeneity in TB trends, with increases observed in parts of Africa and Eastern Europe, while incidence remained stable or declined in other regions. Within this context, Turkey belonged to the group of countries that experienced a declining TB incidence during this period, based on national surveillance data. Since 2000, TB incidence in Turkey has declined more rapidly than the global average, although substantial heterogeneity in TB trends persists across countries, reflecting differences in the effectiveness of TB control programmes and underlying social determinants of health.^[17,25,26]^ These variations underscore the importance of examining country-specific determinants to better understand national TB dynamics.

Turkey’s recent socioeconomic challenges, including financial fluctuations and mass migration, make the country a compelling case for understanding the dynamics of TB incidence rate determinants. Tuberculosis is a disease that encompasses both medical and social dimensions, and TB incidence is closely linked to these factors.^[4]^ This study found that HDI is associated with TB incidence rates in Turkey. In an early study, Dye et al evaluated the determinants of TB incidence rate trends between 1997 and 2006 and found HDI, access to improved sanitation and under-5 mortality rate as dominant predictors at the global level.^[6]^ Költringer et al conducted a similar study from 2005 to 2015, examining 13 social determinants, and found that HDI was associated with TB incidence rates in low- and lower-middle-income countries, while the prevalence of diabetes was associated with TB incidence rates in high- and upper-middle-income countries.^[5]^ Bai and Ameyaw, in their recent study, assessed the main risk factors for TB incidence from 2000 to 2021 using 8 indicators. This research is particularly important for identifying primary and secondary risk factors at the national level. In the mentioned study, the leading risk factor affecting the largest number of countries was the literacy rate, which was also identified as the primary risk factor for Turkey.^[4]^ However, unlike the previous 2 studies, Bai and Ameyaw’s research did not include HDI among the evaluated factors. The HDI is a composite index calculated using scores from 3 key dimensions of human development: life expectancy, education, and gross national income, and it serves as a critical indicator that provides a more comprehensive assessment.^[22]^ During the analyzed period, increases in HDI in Turkey were associated with declines in TB incidence rates. This finding suggests that, despite the fact that countries face notable shifts in various indicators, broader socioeconomic and educational contexts may be relevant to TB incidence trends, alongside medical-focused approaches.

Tuberculosis is widely recognized as a disease closely linked to poverty.^[27]^ Among the 2 key poverty-related indicators, a decrease in GDP per capita and an increase in the poverty headcount ratio were significantly associated with higher TB incidence rates in univariate analysis. Due to their strong correlation, HDI – a more comprehensive measure with greater explanatory power as observed in the univariate analysis – was included in the multivariable analysis instead of these variables. Nevertheless, the potential impact of these 2 parameters on TB control should not be overlooked. It should be noted that retaining HDI while excluding individual economic indicators and year may lead to the concentration of the overall effect within a single variable, and therefore the findings should be interpreted with caution.

Inflation of consumer prices is an economic issue of particular importance for Turkey, characterized by significant fluctuations during the study period.^[23]^ Nevertheless, this variable did not show a significant association with TB incidence in Turkey. An increase in current health expenditure per capita was found to be associated with lower TB incidence rates in the univariate analysis; however, to avoid multicollinearity, this variable was not included in the multivariable model. Although the proportion of the population spending more than 10% of household consumption or income on out-of-pocket health care expenditure showed an association with TB incidence in the univariate analysis, this relationship disappeared in the multivariable analysis.

Turkey’s population has grown rapidly from approximately 65 million in 2000 to over 85 million, and substantial migration driven by regional dynamics has led to significant demographic shifts. However, in our study, population growth rate and net migration did not emerge as determinants of TB incidence rates. The civil war in Syria, Turkey’s southern neighbor, which began in 2011, forced millions of Syrians to seek refuge in neighboring countries. It is well-known that wars and resulting population movements cause a substantial increase in TB risk, not limited to the countries where the conflict occurs.^[28]^ Turkey, which hosts the largest number of Syrian refugees, has seen a rise in imported cases following the Syrian crisis.^[29]^ Despite this, it is important to note that the TB incidence rate has continued to decline across the country. This may be due to the clustering of cases within specific, disadvantaged subgroups. When considered alongside an increase in imported cases, it appears that migration may have slowed the overall decline in TB incidence across the country. Nevertheless, factors like HDI have remained associated with declining TB incidence rates, despite these demographic changes. Evaluating the long-term effects of these demographic shifts will remain important in future research.

While the BCG vaccination rate showed significant association with TB incidence rates in univariate analysis, it was not identified as a determinant of TB incidence in the multivariable analysis. It is well established that the BCG vaccine has a limited effect on preventing the transmission of TB in the community and is primarily effective in protecting against more severe forms of the disease.^[30]^ Additionally, while improved diagnosis and treatment through national TB programs have been associated with lower TB mortality rates, their impact on TB incidence remains uncertain.^[5]^ The lack of a relationship between TB-related health services and incidence aligns with the literature; however, continuing these services is crucial for reducing the overall burden of TB.

To further explore the potential influence of time on the observed associations, sensitivity analyses were conducted. When year-to-year changes were examined, no statistically significant relationships were observed, suggesting that short-term fluctuations in tuberculosis incidence are not strongly related to concurrent annual changes in the examined variables. The similarity of effect directions and magnitudes after adjustment for broad time periods indicates that the main findings are stable over time and are unlikely to be driven by period-specific temporal effects. In this context, and consistent with the sensitivity analyses, the multivariable model should be interpreted as capturing long-term population-level movements rather than isolating short-term or independent effects of individual variables.

This study has several limitations. First, due to its ecological design, the findings are subject to the risk of ecological fallacy, and the observed associations at the population level cannot be directly extrapolated to individual-level risk factors. Second, the lack of reliable and standardized data on some potential determinants, such as diabetes prevalence and HIV prevalence, during the study period restricted their inclusion in the analysis. Third, substantial multicollinearity among HDI, economic indicators, and calendar year may have influenced the estimation of independent associations, and these results should therefore be interpreted with caution. Fourth, the limited number of observations in the longitudinal dataset constrains statistical power. Finally, due to the ecological design and country-specific context, caution is warranted when considering generalization of these findings beyond Turkey. Despite these limitations, the study has notable strengths, including its focus on a country with a large population and its comprehensive evaluation of a broad set of socioeconomic, demographic, and health-related variables, some of which exhibited trends diverging from global patterns.

## 5. Conclusion

In conclusion, the tuberculosis incidence rate in Turkey has substantially decreased over the past 4 decades. Despite fluctuations in other potential TB-related indicators, the Human Development Index showed a consistent association with TB incidence in Turkey. These findings suggest that the decline in TB incidence in Turkey has been observed alongside broader improvements in human development, as captured by the composite HDI. In this context, integrated consideration of HDI components in understanding long-term TB trends may be relevant for informing future research and broader policy discussions related to TB control strategies.

## Author contributions

**Conceptualization:** Yusuf Demirtaş, Turgut Şahinöz.

**Data curation:** Yusuf Demirtaş, Turgut Şahinöz.

**Formal analysis:** Yusuf Demirtaş, Turgut Şahinöz.

**Investigation:** Yusuf Demirtaş, Turgut Şahinöz.

**Methodology:** Yusuf Demirtaş, Turgut Şahinöz.

**Visualization:** Yusuf Demirtaş.

**Writing – original draft:** Yusuf Demirtaş, Turgut Şahinöz.

**Writing – review & editing:** Yusuf Demirtaş, Turgut Şahinöz.

## Supplementary Material


