# Supplementary material for: Trends and determinants of tuberculosis incidence in Turkey: A secondary data analysis
Source: Medicine (Baltimore). 2026 Apr 17;105(16):e48278. doi: 10.1097/MD.0000000000048278 (PMC13095312; doi:10.1097/MD.0000000000048278)
Supplement: Supplementary file 1 [file medi-105-e48278-s001.pdf]

Supplemental Digital Content 1. Table that presents the results of multivariable regression analysis using first-differenced tuberculosis incidence rates and independent variables.

**Variable**	**B**	**95% CI for B**		***p***
Δ Human development index	-0.887	-2.717	+0.943	0.312
Δ Inflation of consumer prices	-2.23×10^{-4}	-0.002	+0.002	0.792
Δ Out-of-pocket HE >10% of income	+0.002	-0.020	+0.023	0.880
Δ BCG vaccination rate	+0.002	-0.002	+0.007	0.251

Δ indicates first-difference transformation (year-to-year change)

HE, health expenditure; BCG, bacille Calmette-Guerin

Supplemental Digital Content 2. Table that presents the results of multivariable regression analysis including a period indicator variable.

**Variable**	**B**	**95% CI for B**		***p***
Human development index	-2.345	-2.869	-1.821	<0.001
Inflation of consumer prices	-0.001	-0.002	+0.001	0.215
Out-of-pocket HE >10% of income	+0.019	-0.003	+0.042	0.078
BCG vaccination rate	+0.003	-5.5×10^{-5}	+0.006	0.054
Period indicator	+0.027	-0.026	+0.081	0.289

Period indicator coded as 0 = 2000-2010 and 1 = 2011-2021

HE, health expenditure; BCG, bacille Calmette-Guerin
